# Identification, validation and candidate gene analysis of major QTL for Supernumerary spikelets in wheat

**DOI:** 10.1186/s12864-024-10540-7

**Published:** 2024-07-08

**Authors:** Zhiqiang Wang, Haojie Li, Xinjian Zhou, Yuzhou Mou, Ying Zhang, Lang Yu, Xudong Chen, Fangkun Wu, Hong Zhou, Yu Lin, Caixia Li, Yaxi Liu

**Affiliations:** 1 State Key Laboratory of Crop Gene Exploration and Utilization in Southwest China, Triticeae Research Institute, Sichuan Agricultural University, Wenjiang, Chengdu, 611130 China; 2 Chengdu Agricultural College, Chengdu, 611130 Sichuan China

**Keywords:** Wheat, QTL, BSE-seq, Supernumerary spikelets

## Abstract

**Background:**

The number of spikelets per spike is a key trait that affects the yield of bread wheat (*Triticum aestivum* L.). Identification of the QTL for spikelets per spike and its genetic effects that could be used in molecular assistant breeding in the future.

**Results:**

In this study, four recombinant inbred line (RIL) populations were generated and used, having YuPi branching wheat (YP), with Supernumerary Spikelets (SS) phenotype, as a common parent. QTL (*QSS.sicau-2 A* and *QSS.sicau-2D*) related to SS trait were mapped on chromosomes 2 A and 2D through bulked segregant exome sequencing (BSE-Seq). Fourteen molecular markers were further developed within the localization interval, and *QSS.sicau-2 A* was narrowed to 3.0 cM covering 7.6 Mb physical region of the reference genome, explaining 13.7 − 15.9% the phenotypic variance. Similarly, the *QSS.sicau-2D* was narrowed to 1.8 cM covering 2.4 Mb physical region of the reference genome, and it explained 27.4 − 32.9% the phenotypic variance. These two QTL were validated in three different genetic backgrounds using the linked markers. *QSS.sicau-2 A* was identified as *WFZP-A*, and *QSS.sicau-2D* was identified a novel locus, different to the previously identified *WFZP-D*. Based on the gene expression patterns, gene annotation and sequence analysis, *TraesCS2D03G0260700* was predicted to be a potential candidate gene for *QSS.sicau-2D*.

**Conclusion:**

Two significant QTL for SS, namely *QSS.sicau-2 A* and *QSS.sicau-2D* were identified in multiple environments were identified and their effect in diverse genetic populations was assessed. *QSS.sicau-2D* is a novel QTL associated with the SS trait, with *TraesCS2D03G0260700* predicted as its candidate gene.

**Supplementary Information:**

The online version contains supplementary material available at 10.1186/s12864-024-10540-7.

## Introduction

Wheat (*Triticum aestivum* L.) is one of the most important staple crops in the world, and estimates suggest that wheat production needs to increase 70% to fulfill food demand [1]. Therefore, maintaining food security and increasing yields to meet the higher demand of a growing global population has become a top priority for breeding programs [2].

Thousand gain weight (TGW), grain number per spike (GNS) and spike number per unit area are three major components of grain yield [3]. Among the above factors, the grain number per spike is affected by inflorescence architecture [4–6]. Plant inflorescences are small florets arranged on an axis, which can be divided into many types, such as racemes, spikes, corymbs, panicles etc. [7]. The wheat inflorescence is a spike, that usually consists of sessile spikelets arranged in opposite rows along the spike axis, each producing 3–5 florets [8]. Therefore, inflorescences, which determine the classification of Gramineae and the yield of food crops, have long been the subject of study of botanists and breeders [9]. In wheat germplasm resources, Supernumerary Spikelets (SS) is a special germplasm with many flowers and grains, which has the potential to improve the yield per unit area in wheat [10, 11]. SS can be divided into two types. One kind is called branched spikelets, where a spare spike extends on the stem node of the original spikelets and spikelets are born on it. The other type of spikelets is that the stem node does not extend, and two or more lateral spikelets are attached to the spike node, which is called supernumerary spikelets [12]. Identification of the QTL for spikelet number per spike (SNS) and its genetic effects that could be used in molecular assistant breeding in the future.

SNS is a complicated trait and controlled by quantitative trait loci (QTL) in wheat. To comprehend the molecular mechanisms that control this trait and to utilize it in breeding, QTL analyses have been conducted in wheat [13, 14]. Six QTL on chromosomes 1 A, 2D, 3B, 6 A, 7 A, and 7D, were found to have dominant and epistatic effects [13]. Cui (2015) detected three QTL on chromosomes 2 A, 5 A, and 7B for SNS that were significant across multiple environments in two recombinant inbred lines (RILs) populations [15]. Four genomic regions affecting SNS on chromosomes 1 A, 1B, 3 A, and 7 A were detected via a population of 191 F_9_ RIL was developed from a cross of two winter cultivars Yumai8679 with Jing 411 [16]. Genotyping-by-sequencing (GBS) and the iSelect 9 K assay were used on a doubled-haploid (DH) soft red winter wheat population that showed a wide range of phenotypic variation for spike traits, and a major QTL *QSl.cz-1 A*/*QFsn.cz-1 A* which explained up to 30.9% of the phenotypic variation for spike length (SL) was identified [17]. In addition, *QFSN4B.4–17* responsible for SNS in multiple environments using a RIL population of 173 lines derived from a cross of the common winter wheat lines Shannong 01–35 and Gancheng 9411 was also identified previously [18].

To date, several genes affecting inflorescence structure and SNS have been identified and designated. For example, the HvMADS1 can directly regulate the expression of *HvCKX3* by binding to the promoter through a CArG-box domain, which affect the cytokinin homeostasis and inflorescence structure in barley [19]. In rice, APO1 interacts with APO2 to synergistically control cell proliferation in meristem and regulates panicle formation, and the overexpression of *APO1* or *APO2* has been associated with significantly increasing the spikelet number [4, 20, 21]. Several genes related to SNS were identified in wheat, including *WFZP* [22], *Ppd-1* [23], *FT2* [24], *WAPO1* [25], *Q* gene [26], and *Grain Number Increase 1* [27].

Although there are many reports about QTL related to wheat spikelet number, only few of QTL have been genetically verified, offering a foundation for fine mapping and map-based cloning. This has greatly restricted the dissection of the molecular mechanisms underlying spikelet number as well as improvement of spikelet number in wheat breeding. Thus, the identification and validation of novel QTL/genes for spikelet number is important. In this study, we used a RIL population (YC) derived from a cross between YuPi branching wheat (YP) and Chinese spring wheat (CS), to identify QTL associated with SS trait using a high-density genetic map and phenotypic data obtained from a multiple environmental trial, in different genetic backgrounds.

## Materials and methods

### Plant materials

The common parent YP is a bread wheat germplasm with the SS character [28, 29]. An RIL population generated by the single seed descent method from a cross YP × CS (YC population, 215 F_6_/F_7_ lines), was used for BSE-Seq and QTL mapping. Three RIL populations derived from the crosses YP × CM107 (YCM population, 132 F_6_/F_7_ lines), YP × CM104 (YJ population, 60 F_6_/F_7_ lines) and YP × 11N21 (NY2 population, 73 F_6_/F_7_ lines), were used to validate the QTL in different genetic backgrounds.

### Phenotypic evaluation and statistical analysis

YC RIL population were planted in three environments: Chongzhou (CZ, 103° 38′ E, 30° 32′ N) in 2021–2022 (2022CZ), Chongzhou and Wenjiang (WJ, 103° 41′ E, 30° 36′ N) in 2022–2023 (2023CZ and 2023WJ). A randomized complete block design was adopted in all environments. YCM, YJ and NY2 populations were planted at Chongzhou in 2022–2023. Fifteen seeds of each family were planted in 1.5 m rows spaced 30 cm apart. Field management was performed according to recommendations for wheat production. After maturity, five plants from the center rows were selected and used for phenotypic evaluation, including the number of SS per spike (NSS), SNS, TGW, GNS, grain weight per spike (GWS), grain length (GL), grain width (GW), flag leaf length (FLL) and flag leaf width (FLW). The SNS was determined by counting the total spikelets in the main spike, NSS was calculated by deducing the number of nodes per spike from SNS. GNS, GWS, GL and GW were measured by a Wanshan SC-G automatic seed test system (Hangzhou Wanshen Detection Technology Co., Ltd., CN). The TGW was calculated as 10-fold of the weight of 100 seeds measured with an electronic balance, in three replicates for each line [1].

Analysis of variance (ANOVA) for the NSS in each trial and Pearson correlations between variables were computed in SPSS v27 (IBM SPSS, Chicago, IL, USA). To minimize environmental effects, the best linear unbiased estimates (BLUE) for NSS in three replicates was calculated using SAS 8.1 (SAS Institute Inc., Cary, NC, USA). According to the method described by Smith, broad-sense heritability (*h*^*2*^) across environments was estimated, according to the equation *h*^*2*^ = *V*_*G*_/(*V*_*G*_ + *V*_*GE*_*/r* + *V*_*E*_), where *V*_*G*_ = genotypic variance, *V*_*GE*_ = genotype × environment variance, *r* = number of replicates, and *V*_*E*_ = environmental variance. Student’s *t*-tests (*P* < 0.05) were applied to compare lines classified by genotype [30, 31].

### Bulked Segregant Analysis and Exome sequencing

Total genomic DNAs were isolated from young leaves of the tested cultivars and RILs using a CTAB method followed by RNase-A digestion [32]. Isolated DNA was quality checked by resolving on a 1% agarose gel electrophoresis and concentration was determined using a UV spectrometer [33, 34]. Two DNA pools were constructed by mixing equal amounts of DNA normalized to the concentration 100 ng/µL from 30 extreme SS (SS-pool) and 30 normal spike types (NS-pool), respectively. The DNA libraries were constructed through DNA fragmentation, end-repair, adaptor ligation, PCR and hybridization capture as previously described [35]. Curated sequence data was aligned to the CS reference genome sequence (RefSeq) v2.1 by BWA software [36]. The BCFtools software was used to detect and extract the single nucleotide polymorphism (SNP) and InDel [37]. The SNP and InDel were annotated using ANNOVAR, which mainly included different regions of the genome and different types of exon regions [38]. SNP-index methods were used to screen the SNP and InDel sites with candidate regions between the progeny mixed pools in this study [39–42]. To identify candidate regions associated with the SS trait, the ΔSNP-index of each locus was calculated by subtracting the SNP-index of the SS-pool from that of the NS-pool according to previous method [43]. To confirm the results of ΔSNP-index, an algorithm was further performed to identify the SNPs and InDels associated with the SS trait using the equation reported previously [31, 40], and the absolute value of Δ(SNP-index) was used for Locally Weighted Scatterplot Smoothing (LOESS) to obtain the correlation threshold [2]. The greater the ΔSNP-index, the more likely the SNPs and InDels contribute to the trait of SS or is linked to a locus that controls the trait.

### Marker development, genetic map construction and QTL identification

To validate the BSE-seq results and further narrow down the region, primers were designed to the flank the sequences of the targeted SNPs using an online tool of the wheatomics platform (http://wheatomics.sdau.edu.cn/PrimerServer/). Polymorphic SNPs between YP and CS in the initial mapping region were converted to KASP markers following previously described methods [44]. The PCR for InDel primers was conducted in a 20 µL reaction volume with 1.0 µM primer mix, 2.5 ng/µL DNA and 1× Taq Master Mix (Vazyme Biotech, CN). PCR was performed using routine procedures, and the polyacrylamide gel (8%) electrophoresis was used for resolving amplified products [34]. The polymorphisms of the markers were confirmed by parental liens and some progenies.

Linkage mapping was conducted using JoinMap v4.0 [45]. The maximum likelihood mapping algorithm and Kosambi’s function were used to determine the marker order and distance, respectively. QTL analysis of SS trait was performed by Interval Mapping (IM) with the software MapQTL v5.0 [46]. For each trial, a test of 1,000 permutations was performed to identify the LOD threshold corresponding to a genome-wide false discovery rate of 1% (*P* < 0.01).

### QTL validation

For QTL validation, the NSS from all homozygous lines in each of the three RIL populations (YCM, YJ and NY2) were counted. Based on marker profiles, individuals in each population were grouped into two classes as described above, and the difference in the average spikelet number between these classes was used for measuring the QTL effects within each validation population. The Student’s t-test was used to determine the significance in differences between the two groups in each population at *P* < 0.05.

### Prediction of candidate genes for QTL

The physical interval of QTL was obtained by conducting a homolog search of the flanking makers against the Chinese Spring reference genome (IWGSC_Refseq v2.1), and the genes mapped to the region were identified. Further, information on the genes were obtained from the wheatomics platform (http://wheatomics.sdau.edu.cn/). The expression values as transcripts per million (TPM) in roots, stems, leaves, spikes, and grain were obtained from the GeneExpression of Wheatomics platform. Genes were annotated through BLAST against the corresponding protein sequences in rice and *Arabidopsis thaliana* on KOBAS v3.0 [47]. The total RNA was extracted by using the RNeasy plant mini kit (Qiagen, CN) from CS and YP at the correct stage, and the products after reverse transcription were used for quantitative RT-qPCR. The genomic DNA of parents was used to amplify candidate genes for sequence analysis.

## Results

### The NSS of YC RIL population in different environments

**Fig. 1:**
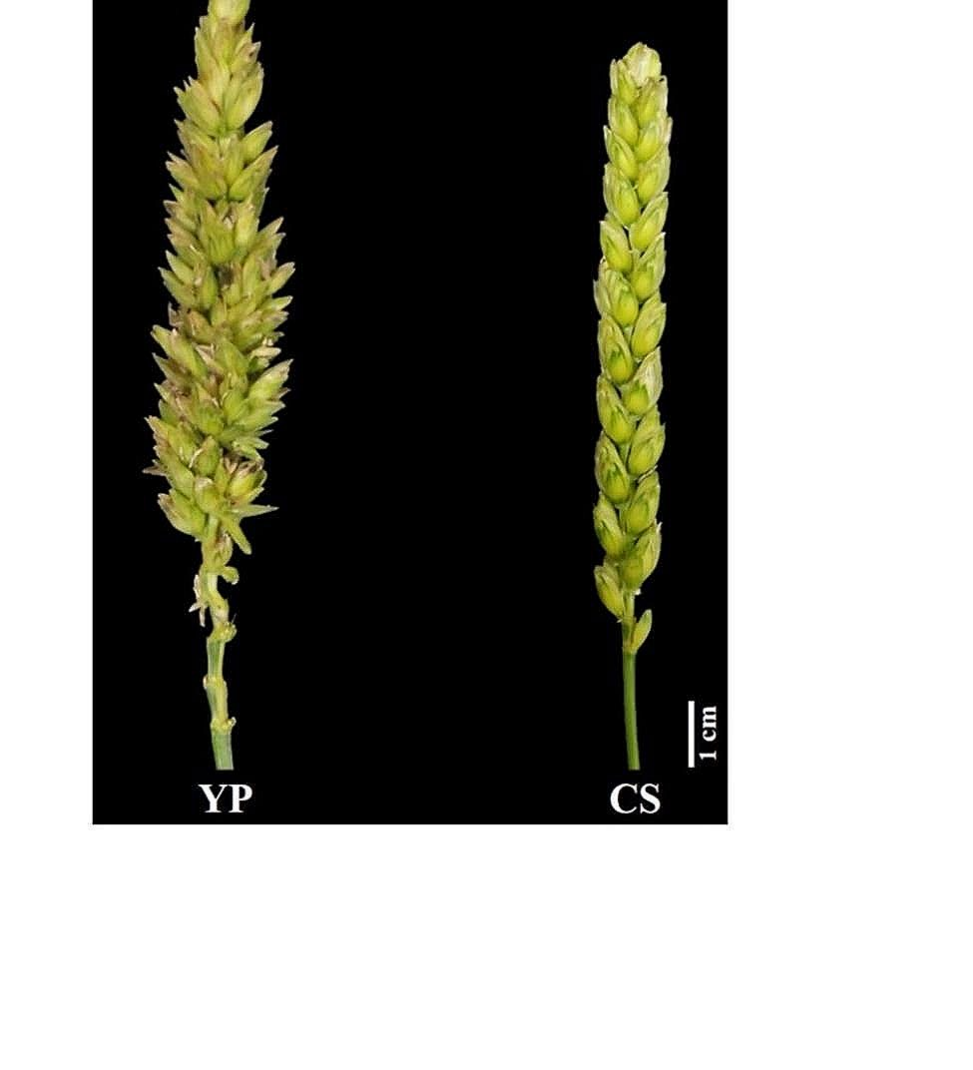
Spike features of YuPi branching wheat (YP) and Chinese spring wheat (CS)

In the different environments, the NSS of CS remained stable at 0, while that of YP ranged from 56.4 to 65.8, with the NSS of YP being significantly (*P* < 0.01) higher than that of CS (Table 1; Fig. 1). The NSS trait in YC population was also observed in different environments, ranged from 0 to 63.6, and the broad-sense heritability was estimated as 0.96 (Table 1). The correlation coefficients between the different environments were all significant and ranged from 0.922 (*P* < 0.01) to 0.982 (*P* < 0.01) (Table 2). The BLUE values of SNS, GWS, TGW, GNS, GL, GW, FLL and FLW were shown in Table 1 and were used to assess the effect of SS QTL on these traits.

**Table 1:** Distribution of NSS in the YC population

Trait	Environment	Parents		Population				
		Mean of YP	Mean of CS	Range	Mean		SD	H^2^
NSS	CZ2022	56.40**	0.00	0.00-36.60		5.00	8.10	
	WJ2023	65.80**	0.00	0.00-63.60		7.81	14.51	
	CZ2023	62.20**	0.00	0.00-58.20		5.82	10.91	
	BLUE	57.31**	0.21	0.21-53.0		6.42	10.74	0.96
SNS	BLUE	70.51**	26.82	16.94–73.55		30.17	10.36	0.88
GWS	BLUE	3.87**	1.91	1.52–2.93		2.01	0.21	0.55
TGW	BLUE	37.49**	27.92	23.38–43.10		34.10	3.78	0.76
GNS	BLUE	108.07**	62.71	41.56–85.34		59.33	7.94	0.62
GL/mm	BLUE	6.82**	5.24	6.54–9.16		7.97	0.40	0.87
GW/mm	BLUE	2.78	2.72	3.23–4.17		3.79	0.17	0.74
FLL/cm	BLUE	22.61**	25.12	18.20-29.54		22.49	2.04	0.70
FLW/cm	BLUE	1.84**	1.53	1.27-2.00		1.60	0.12	0.72

** indicate significant differences at P *<* 0.01. NSS, the number of SS per spike. SNS, spikelets number per spike. GNS, grain number per spike. GWS, grain weight per spike. TGW, thousand gain weight. GL, grain length. GW, grain width. FLL, flag leaf length. FLW, flag leaf width. BLUE, best linear unbiased estimator

**Table 2:** Correlation coefficients for the NSS in the YC population

repetition	CZ2022	CZ2023	WJ2023	BLUP
CZ2022	1			
CZ2023	0.922^**^	1		
WJ2023	0.922^**^	0.925^**^	1	
BLUE	0.965^**^	0.982^**^	0.973^**^	1

** indicate significant differences at *P* < 0.01

### BSE-seq analysis

The results obtained from the exome capture sequencing of bulked segregants were compared with the CS reference genome v2.1. We use the ΔSNP-index algorithm to calculate the allele segregation of the SNPs and InDels between two extreme DNA pools. ΔSNP-index algorithm showed abundant candidate SNPs/InDels enriched on chromosome 2 A and 2D (Chr2A and Chr2D). ΔSNP-index greater than 95% confidence interval was selected as the threshold for screening [48]. A 16.3 Mb region of Chr2A (63.8–80.1 Mb) and a 9.1 Mb region of Chr2D (68.1–77.2 Mb) were identified as the candidate regions for SS trait (Figure S1).

### Linkage map construction and QTL identification

**Fig. 2:**
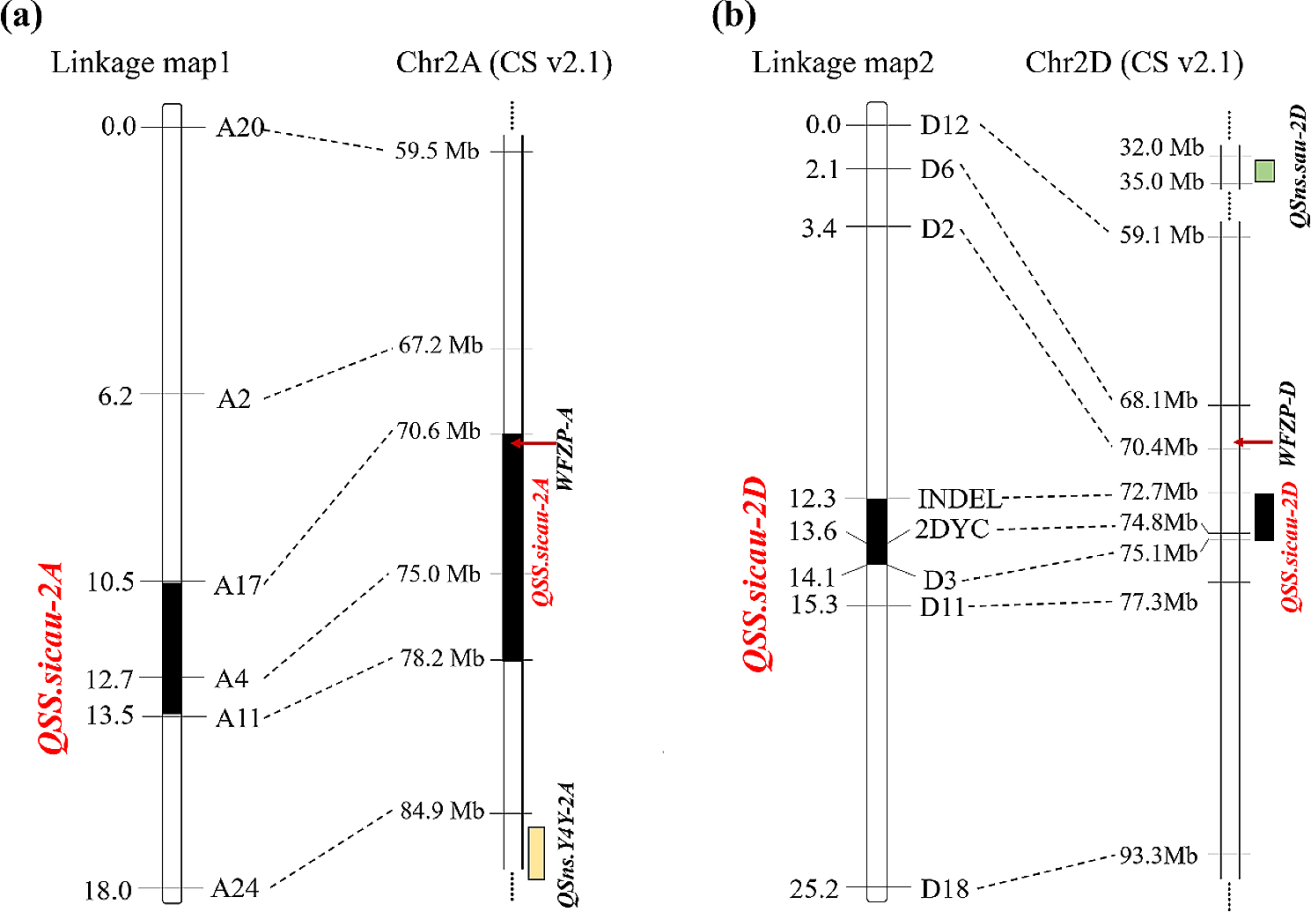
Linkage map of chromosome 2 A and chromosome 2D. (**a**) Physical map of chr2A, with *QSS.sicau-2 A* mapped on a region of the 3.0 cM genetic map. (**b**) Physical map of Chr2D, with *QSS.sicau-2D* mapped on a region of the 1.8 cM genetic map

To confirm the preliminarily identified genomic regions responsible for SS trait, SNPs and InDels in the target regions were converted into KASP and InDel markers, and were used for the construction of the genetic map. In total, 13 KASP markers and one InDel marker were used for the construction of the genetic maps. Six KASP markers developed for Chr2A generated a linkage map spanning 18.0 cM, while seven KASP markers and one InDel marker developed for Chr2D generated a linkage map spanning 25.2 cM (Fig. 2, Table S1). The phenotypic data of SS trait evaluated in the three environments were used for QTL mapping. Two stable QTLs named *QSS.sicau-2 A* and *QSS.sicau-2D* were detected in three environments and BLUE. *QSS.sicau-2 A* located between A11 and A17, explained 13.7–15.9% of the phenotypic variance with the LOD values ranging from 6.9 to 8.1. *QSS.sicau-2D* located between INDEL and D3, explained 27.4–32.9% of the phenotypic variance with the LOD values ranging from 15.0 to 18.7 (Table 3). The favorable alleles of the two QTL were all contributed by YP.

**Table 3:** QTL analysis for SS in different environments and the BLUE datasets

QTL	Environment		Flanking markers	LOD	LOD Thre	PVE(%)	Add	Physical interval(Mb)
*QSS.sicau-2 A*		CZ2022	A11, A17	7.9	3.2	15.5	3.72	70.6–78.2
		CZ2023	A11, A17	8.0	3.3	15.7	6.44	70.6–78.2
		WJ2023	A11, A17	6.9	3.2	13.7	4.62	70.6–78.2
		BLUE	A11, A17	8.1	3.4	15.9	4.90	70.6–78.2
*QSS.sicau-2D*		CZ2022	INDEL, D3	15.0	2.9	27.4	6.62	72.7–75.1
		CZ2023	INDEL, D3	15.3	2.9	27.8	8.69	72.7–75.1
		WJ2023	INDEL, D3	18.7	3.1	32.9	5.49	72.7–75.1
		BLUE	INDEL, D3	17.1	3.5	30.6	6.91	72.7–75.1

LOD, logarithm of odds. LOD Thre, the LOD threshold. PVE, phenotypic variation explained. Add. additive effect (positive values indicate that alleles from YP increased trait scores, and negative values indicate that alleles from CS increased trait scores). BLUE, best linear unbiased estimator

### Effects of supernumerary spikelet QTL in different genetic backgrounds

**Fig. 3:**
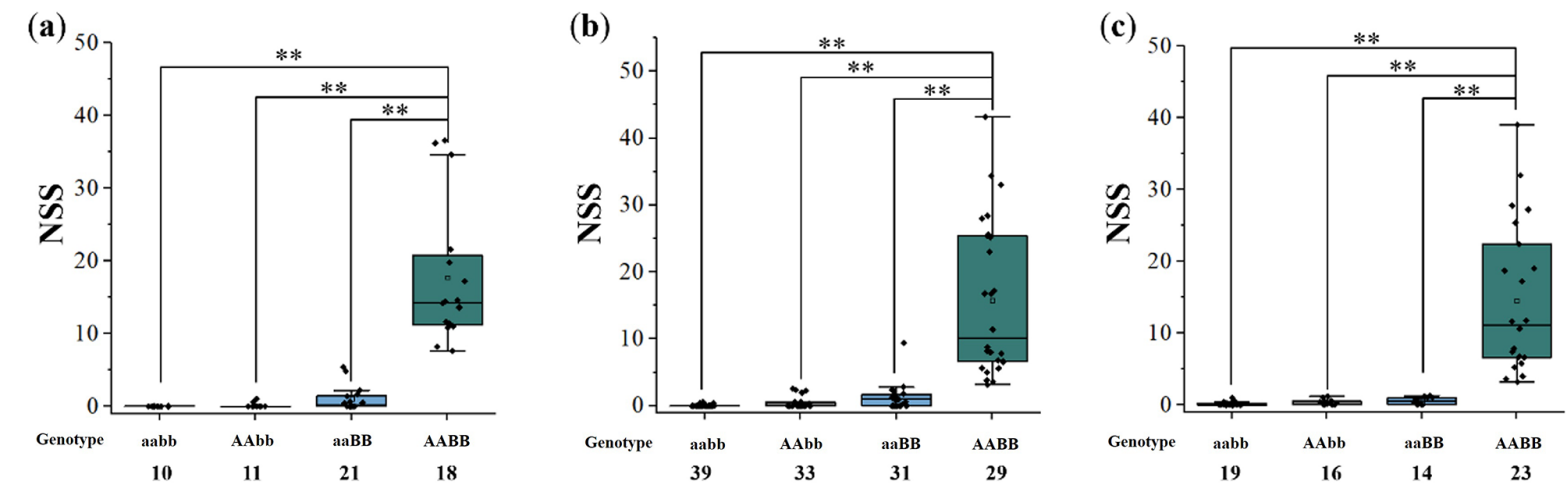
The effects of *QSS.sicau-2 A* and *QSS.sicau-2D* on NSS in the validation populations. (**a**) YP/CM104 RIL population. (**b**) YP/CM107 RIL population. (**c**) YP /11N21 RIL population. ** indicate significance level at *P* < 0.01 by the Student’s t-test. NSS, the number of SS per spike

Three KASP markers (A4, D3, 2DYC) and one InDel marker (INDEL) were used to assess the effects of QTL on NSS in validation populations (A4 is linked to *QSS.sicau-2 A* and exhibits polymorphism across the three validation populations. INDEL, 2DYC and D3 are linked to *QSS.sicau-2D* and exhibit polymorphism in YJ, G2 and NY2 populations, respectively). In YJ population, the average NSS in homozygous “AA, DD” genotype was 17.71, that was significantly (*P* < 0.01) increased than that in homozygous “aa, dd”, “AA, dd” and “aa, DD” genotypes. Similarly, in G2 and NY2 populations, the average NSS in homozygous “AA, DD” genotypes were 15.75 and 14.52, respectively, that significantly (*P* < 0.01) increased than that in other three homozygous genotypes (Fig. 3).

### Candidate gene prediction

According to the CS genome (IWGSC Refseq v2.1), *QSS.sicau-2 A* was located between 70.6 Mb and 78.2 Mb on Chr2A, and 91 high-confidence genes, including *WFZP-A* (*TraesCS2A03G0239400*) spanned the region. Sequence analysis of the *WFZP-A* (Table S2), revealed a 4-bp deletion in *WFZP-A* resulting a frame-shift in YP (Figure S2, Table S3), and the variation type is consistent with Zang734 [49]. These results suggest that *WFZP-A* is likely responsible for *QSS.sicau-2 A.*

**Fig. 4:**
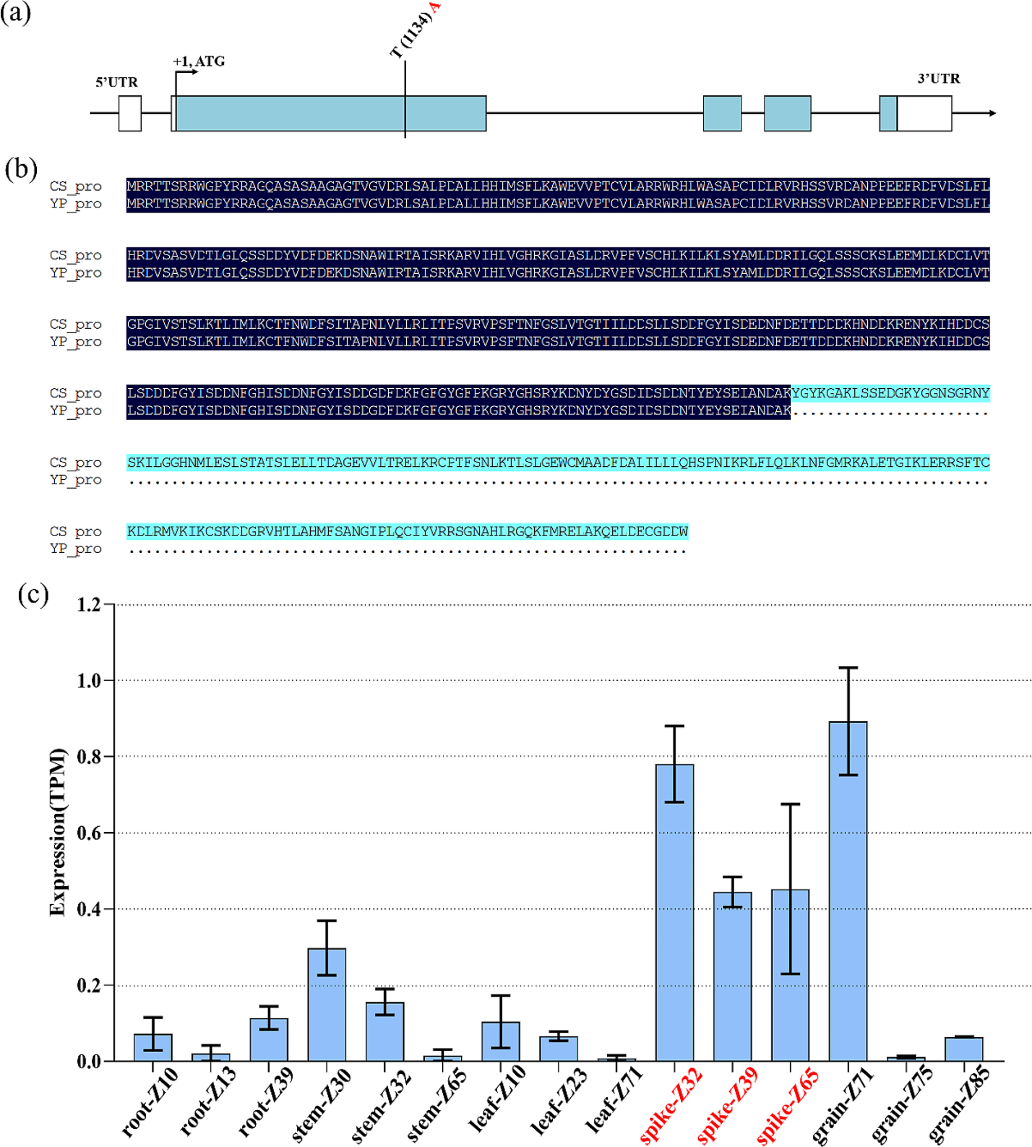
Variation sequence and expression patterns of *TraesCS2D03G0260700*. (**a**) The nucleotide of YP and CS are shown in red and black, respectively. (**b**) The amino acid sequence encoded by the *TraesCS2D03G0260700* in YP and CS. (**c**) *TraesCS2D03G0260700* expression patterns in different tissues, the data (TPM value) were downloaded from the GeneExpression of WheatOmics (http://wheatomics.sdau.edu.cn/). Z10, first leaf through coleoptile. Z13, 3 leaves unfolded. Z23, main shoot and 3 tillers. Z30, pseudo stem erection. Z32, 2nd node detectable. Z39, flag leaf ligule/collar just visible. Z65, anthesis half-way. Z71, caryopsis water ripe. Z75, medium milk. Z85, soft dough

*QSS.sicau-2D* was mapped between 72.7 Mb and 75.1 Mb on Chr2D, revealing 34 high-confidence genes in the region (Table S4). Eight spike/grain-specific genes were identified by gene expression of WheatOmics (http://202.194.139.32/expression/index.html), that might probably be involved in spike growth and development (Table S5, Figure S3). Exome sequencing data of YP reveal one T to A substitution which converts the codon (TAT) to a translational stop codon (TAA) in *TraesCS2D03G0260700* (Fig. 4). Additionally, we examined the coding sequences of the known branching gene *WZFP-D* (*TraesCS2D03G0248500*, chr2D: 69,940,372.69,941,615). No sequence variation between YP and CS was identified for *WZFP-D*, and no significant difference (*P* > 0.05) in gene expression in YP and CS (Figure S4, Table S3).

### Effects of *QSS.sicau-2 A and QSS.sicau-2D* on yield-related traits

**Fig. 5:**
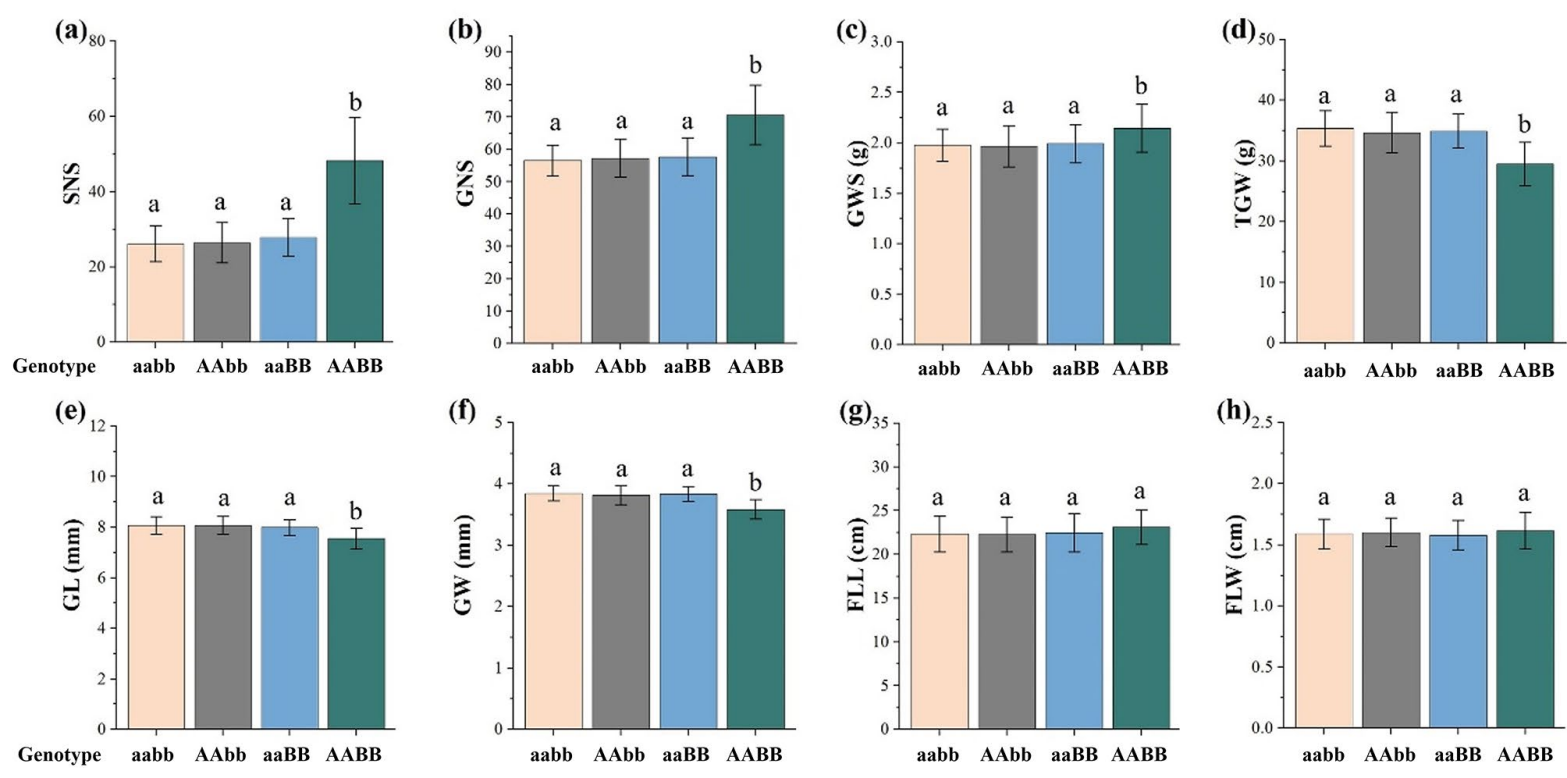
Effects of *QSS.sicau-2 A and QSS.sicau-2D* on grain traits and flag leaf. Bar pattern and significance analysis of (**a**) SNS, (**b**) GNS, (**c**) GWS, (**d**) TGW, (**e**) GL, (**f**) GW, (**g**) FLL and (**h**) FLW. Each bar shows mean ± SD. Different letters indicate significance level at *P* < 0.05 by the Tukey’s test. SNS, spikelets number per spike. GNS, grain number per spike. GWS, grain weight per spike. TGW, thousand gain weight. GL, grain length. GW, grain width. FLL, flag leaf length. FLW, flag leaf width

Two QTL, *QSS.sicau-2 A* and *QSS.sicau-2D*, were identified in YC population. Their effects on the yield-related traits were analyzed by linking markers. The YC population was divided into four genotypes of “aa, dd” “AA, dd”, “aa, DD” and “AA, DD” by molecular markers. The SNS, GNS and GWS of homozygous “AA, DD” genotypes were significantly higher than that of other three genotypes, the TGW, GL and GW of homozygous “AA, DD” genotypes were significantly lower than that of other three genotypes (Fig. 5a and f). Additionally, the FLL and FLW were not affected by *QSS.sicau-2 A and QSS.sicau-2D* (Fig. 5g and h).

## Discussion

The complexity of the wheat genome combined with the fewer spikelet number mutants, hinder the identification of beneficial variants associated with spikelet number. To enhance the yield potential of wheat, breeders have tried to alter its sink capacity by modifying spike morphology [50]. The supernumerary spikelet (SS) character of bread wheat (*Triticum aestivum* L.) is an abnormal spike morphology with extra spikelets per rachis node. In some materials, the SS trait was unstable, and some environmental factors and photoperiod could affect the expression of *bh* gene for SS character [51, 52]. In this study, YP maintained SS trait in multiple environments, which provides an available germplasm resource for improving wheat spikelets.

### Comparison of QTL identified for wheat spikelet number with previous studies

In recent years, many QTLs related to SNS have been widely reported and were mapped to all 21 chromosomes of wheat [14, 53–56]. *QSns.Y4Y-2 A*, a QTL related to SNS, was detected in multiple environments and mapped between 85.6 Mb and 91.1 Mb [57]. Li [58] found one local cultivar YM44 that has SS phenotype, which was linked to the marker *Xwmc522* and *cfd56* mapped on chromosome 2 A and 2D, respectively, and further confirmed that *WFZP-A* is the functional gene of chromosome 2 A that causes SS phenotype. In this study, we found that *WFZP-A* (Chr2A: 71,582,645.71,583,948) exists in the *QSS.sicau-2 A* (70.6–78.2 Mb) interval, and the similar variation existed in YP by the sequences analysis of *WFZP-A* in YP and CS (Figure S2). Therefore, we speculate that *WFZP-A* may be responsible for *QSS.sicau-2 A*.

In addition, several QTL controlling SNS trait have been reported on chromosome 2D in wheat. *QSns.sau-2D* was mapped on chromosome arm 2DS flanked by the markers *AX-109,836,946* (32.8 Mb) and *AX-111,956,072* (34.4 Mb) [59]. Gene *Mrs1* on the short arm of chromosome 2D, that closely linked to *Xgwm 484* (50.6 Mb), and further identified the candidate gene *WFZP-D* (chr2D: 69,940,372.69,941,615) by homologous function annotation [22, 60]. In this study, *QSS.sicau-2D* was mapped to the genomic region of Chr2D between 72.7- and 75.1 Mb, and no overlap between the QTL interval associated with SNS and indicating *QSS.sicau-2D* may be a novel QTL for SS trait.

### Potential candidate genes for *QSS.sicau-2D*

To screen the candidate gene for *QSS.sicau-2D*, we amplified *WFZP-D* in the cDNA of both parents even though *WFZP-D* is not in the interval of *QSS.sicau-2D*. The results showed that *WFZP-D* had no sequence difference between YP and CS. Furthermore, there was no significant difference (*P* > 0.05) in the expression of *WFZP-D* in YP and CS by RT-qPCR analysis (Figure S4). One SNP was identified between CS and YP 60 bp downstream of the *WFZP-D* and a KASP marker named D21 was developed to assay the polymorphism. The D21 marker was mapped 9.4 cM away from *QSS.sicau-2D* in the reconstructed genetic map (Figure S5). These results indicated the *WFZP-D* is not the candidate gene for *QSS.sicau-2D*.

Eight spikelet/grain specific expressed genes were identified in the *QSS.sicau-2D* region, among which, one SNP converts the codon (TAT) to a translational stop codon (TAA) of *TraesCS2D03G0260700* in YP by exome sequencing and Sanger sequencing analysis. In addition, *TraesCS2D03G0260700* encoding cyclin-like F-box domain protein, and previous studies have shown that cyclin-like F-box domain is specifically expressed in wheat inflorescence and involved in the development of wheat inflorescence [61–65]. Overall, *TraesCS2D03G0260700* was predicted to be a potential candidate gene for *QSS.sicau-2D*.

### Effects of *QSS.sicau-2 A and QSS.sicau-2D* on yield-related traits

Consistent with previous studies, GL, GW and TGW have decreased while GNS has increased, with the increase of SNS (Fig. 5), such as a single mutation in *WFZP-D* can significantly increase the SNS and GNS [49, 58, 59]. GWS is an important trait influencing wheat yield [66]. In this study, we found that the GWS exhibited a significant increase in YC population when *QSS.sicau-2 A* and *QSS.sicau-2D* coexisted. This may be attributed to the compensatory increase in grain number per spike (GNS) offsetting the decrease in thousand-grain weight (TGW). In conclusion, the SS trait have the value of increasing the yield potential of wheat.

## Conclusion

In this study, two major SS QTL were identified in multi-environments and validated in different genetic populations. *QSS.sicau-2D* is a new QTL of SS trait, and predicted *TraesCS2D03G0260700* to be its candidate gene. At the same time, the combined effect of *QSS.sicau-2 A* and *QSS.sicau-2D* significantly increased GWS, which has the application value of increasing the yield potential of wheat.

### Electronic supplementary material

Below is the link to the electronic supplementary material.

Supplementary Material 1

Supplementary Material 2

## Data Availability

The datasets generated during the current study are available at the National Center for Biotechnology Information (NCBI) SRA repository under accession number PRJNA1041571.
