# Supplementary material for: Identification, validation and candidate gene analysis of major QTL for Supernumerary spikelets in wheat
Source: BMC Genomics. 2024 Jul 8;25:675. doi: 10.1186/s12864-024-10540-7 (PMC11229243; doi:10.1186/s12864-024-10540-7)
Supplement: Supplementary file 1 — Supplementary Material 1 [file 12864_2024_10540_MOESM1_ESM.docx]

**Supplementary Figure** :


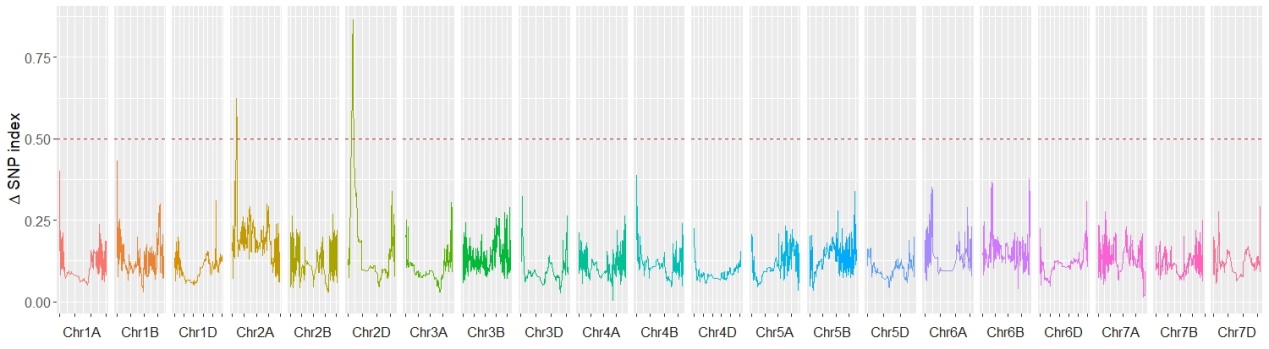


**Figure S1**. The ΔSNP-index chromosomal interval coloring map by BSE-seq analysis. The X-axis represents chromosome of wheat, and Y-axis represents delta ΔSNP-index.


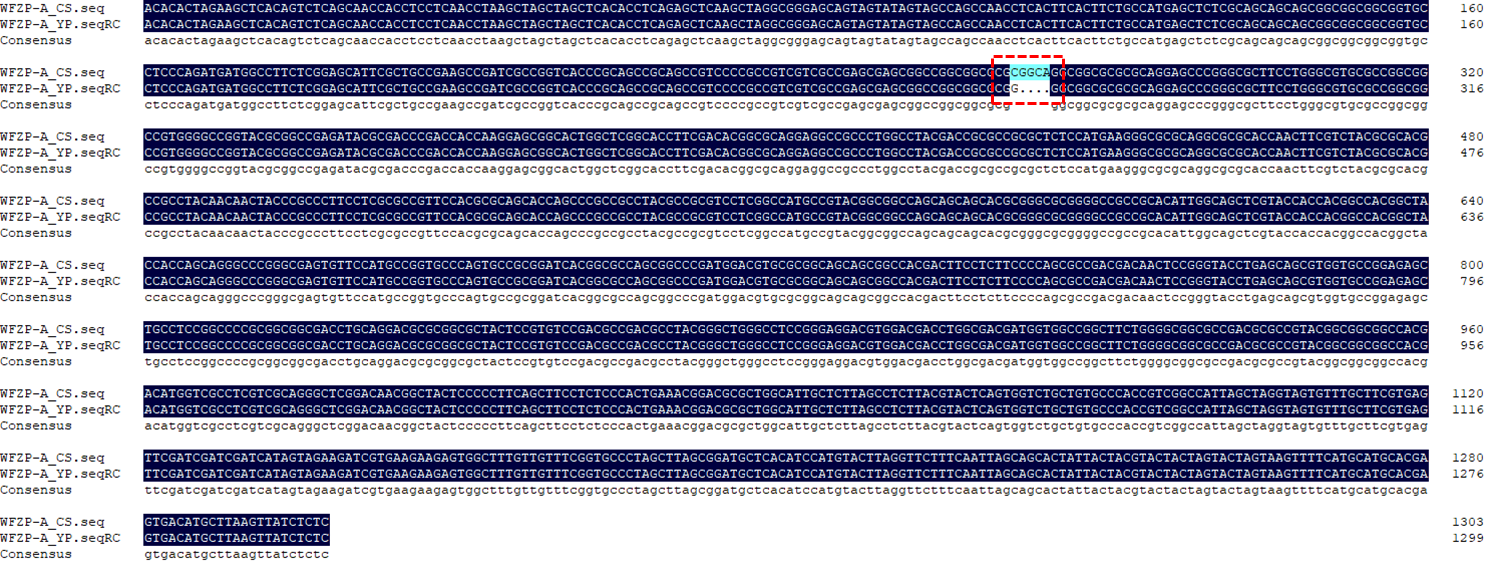


**Figure S2**: Sequence information of *WFZP-A*. The red mark is the mutation type of *WFZP-A* in YP (CGGCA to G).


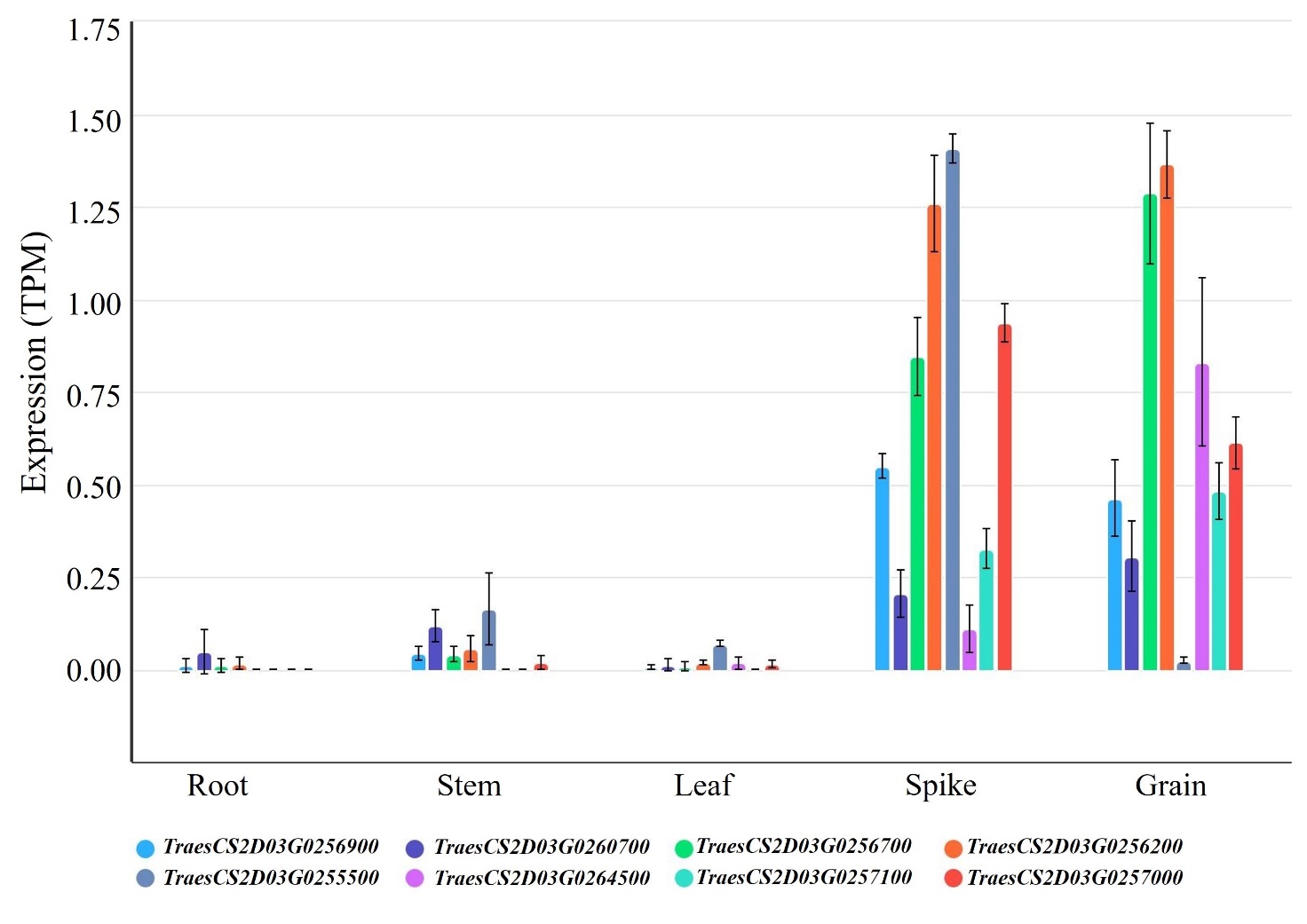


**Figure S3**: Expression patterns of the eight genes in different tissues (roots, leaves/shoots, spike and grain), the data (TPM value) were downloaded from the GeneExpression of WheatOmics (http://wheatomics.sdau.edu.cn/).


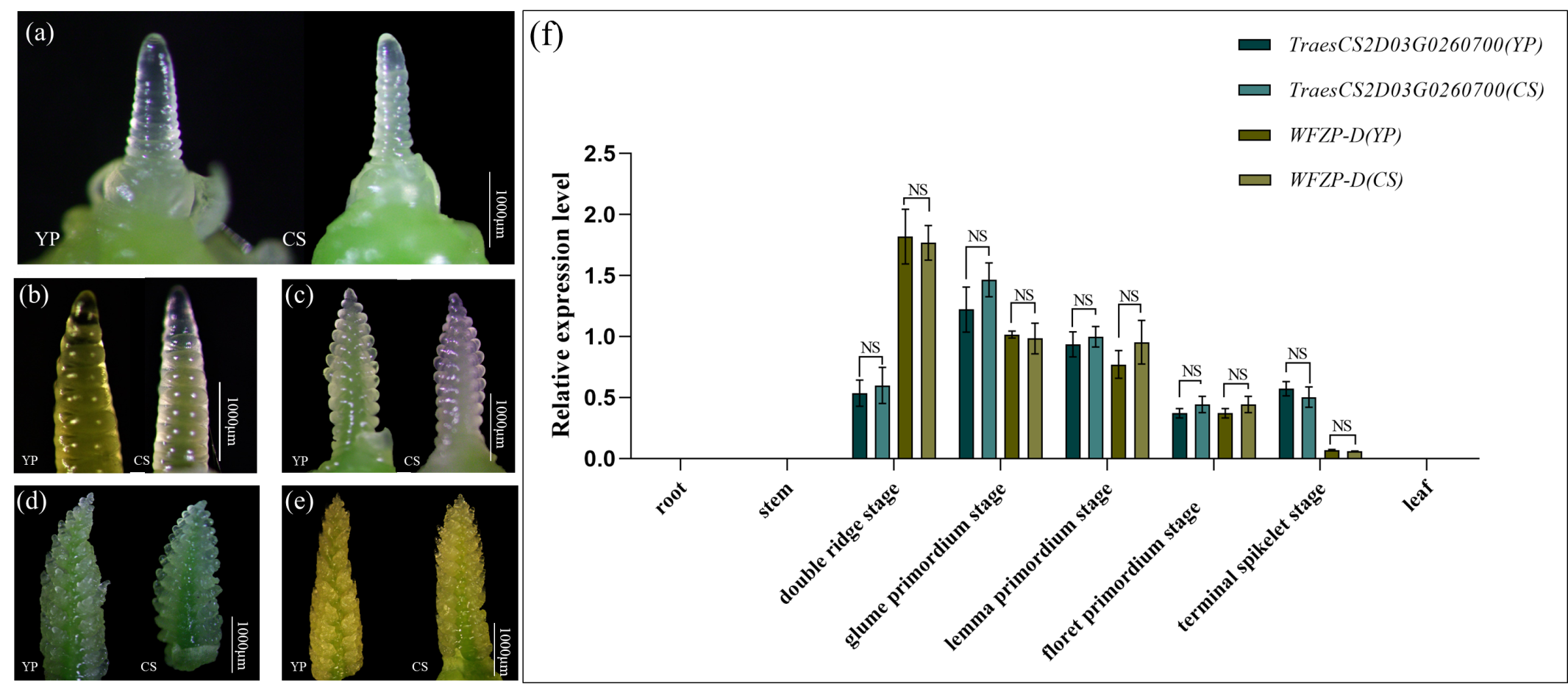


**Figure S4**: The expression analysis of *WFZP-D* and *TraesCS2D03G0260700*. (a) The graphics of CS and YP at double ridge stage. (b) The graphics of CS and YP at glume primordium stage. (c) The graphics of CS and YP at lemma primordium stage. (d) The graphics of CS and YP at floret primordium stage. (e) The graphics of CS and YP at terminal spikelet stage. (f) The expression analysis of *WFZP-D* and *TraesCS2D03G0260700*. NS, indicate not significant differences at P > 0.05.


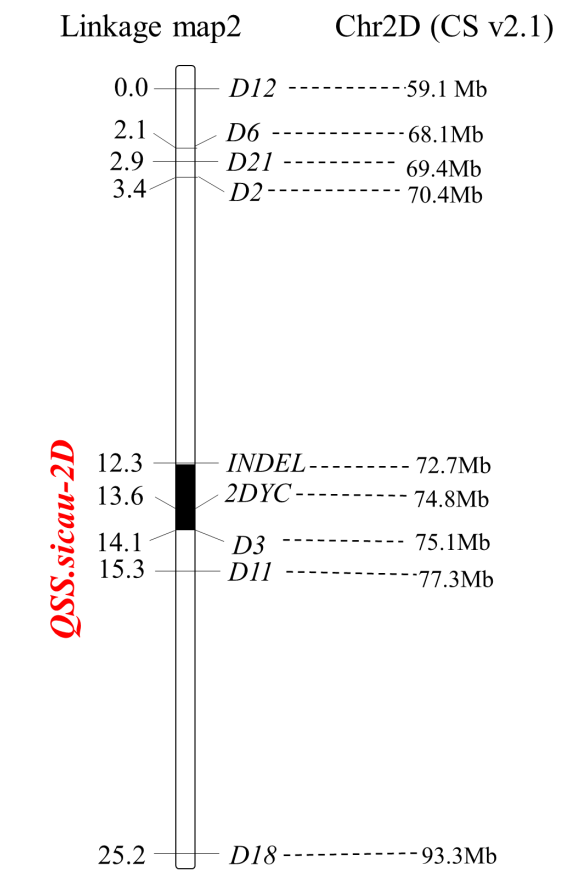


**Figure S5**: The reconstructed genetic map of *QSS.sicau-2D*.
